# “And you feel like you’re suffocating … how the fuck am I going to get out of all this?” Drivers and experiences of suicidal ideation in the Australian construction industry

**DOI:** 10.3389/fpsyt.2023.1144314

**Published:** 2023-05-12

**Authors:** Simon Tyler, Kate Gunn, Bob Clifford, Nicholas Procter

**Affiliations:** ^1^Mental Health and Suicide Prevention Research and Education Group, Clinical and Health Sciences, University of South Australia, Adelaide, SA, Australia; ^2^Allied Health and Human Performance, University of South Australia, Adelaide, SA, Australia; ^3^MATES in Construction South Australia, Adelaide, SA, Australia

**Keywords:** construction industry, suicide, suicidal ideation, mental health, distress, prevention

## Abstract

**Introduction:**

This research was designed to generate understandings of drivers and experiences of suicidal ideation and distress among Australian Construction Industry (ACI) workers, as well as what helped during these experiences.

**Methods:**

Fifteen participants, from a variety of ACI or closely associated roles, with an average age of 45 years (29–66), engaged in individual, semi-structured interviews. Interviews were audio-recorded with consent and analyzed using descriptive thematic analysis.

**Results:**

Eight themes relating to what may drive the presence of suicidal ideation and distress were identified; 1) challenges of working within the ACI, 2) relationship and family issues, 3) social disconnection, 4) personal financial hardship, 5) perceived lack of support, 6) alcohol and drug use, 7) child custody/access and legal issues, and 8) experience of mental health challenges, trauma, or a significant adverse life event. Four themes relating to experience and expression of suicidal ideation and distress were identified: 1) suicidal thoughts, 2) impaired thinking, 3) observable expressions of suicidal distress, and 4) lack of observable expressions of suicidal distress. Six themes relating to what helped during experiences and well as what can be done by the ACI to help mitigate experiences, were identified: 1) presence of colleague and managerial support, 2) MATES in Construction, 3) engagement with non-work activities and social support, 4) personal skills and knowledge relating to suicide and mental health, 5) high level industry integration and engagement with support programs, and 6) work hours and expectations changes.

**Discussion:**

Findings highlight several industry and personal related challenges that may drive experiences, with many potentially mitigatable by ACI changes and focused prevention strategies. Participant suicidal thought descriptions align with previously identified constructs deemed central in suicidal trajectories. While findings highlight several observable expressions of suicidal ideation and distress, challenges associated with identifying and assisting individuals in the ACI who may be struggling were also reported. Several factors that helped ACI workers during their experiences, as well as what the ACI can do to mitigate future experiences, were identified. Recommendations are made based on these findings, encouraging a more supportive work environment, as well as continued development and increased awareness of support and education systems.

## 1. Introduction

While still a developing area of investigation, research has suggested Construction Industry Workers (CIW) are at increased vulnerability to suicide with repeated reporting of rates far greater for CIW than a range of comparison populations (1–3). For example, CIW from the U.S. state of Alabama were shown to be 11 times more likely to die by suicide than public administration workers (4). Additionally, a recent meta-analysis provided further support for this notion, with an increased random effect pooled relative suicide risk for CIW in comparison to general working populations reported (5).

Given the developing nature of the area, reasons for increased CIW vulnerability to suicide remain open for debate. While recent reviews of the topic have shed some light on what may drive these outcomes, they also suggest further research, with a particular focus on understanding drivers from within the industry, is required (6, 7). Additionally, a distinct lack of understanding of what it is like to experience the onset or worsening of suicidal trajectories for CIW, as well as what helped during these challenging times, is apparent (3, 7, 8). This lack of high quality and extensive evidence is likely the result of limited data availability, hampered by a number of factors including low incidence base rates for suicide (9, 10). Similarly, limitations due to low incidence base rates are also likely if endeavouring to understand experiences of suicidality in CIW through investigation of those who have made suicidal attempts. For example, it is regularly reported that men are less likely to attempt suicide in comparison to women (11). With the construction industry heavily male dominated, coupled with the fact men are less likely to engage in health behavior research than women, the ability to generate substantive and quality empirical evidence in the area is limited (12). Additionally, scholarly suggestion is that suicide morbidity and attempts are the “tip of the iceberg” regarding suicidal trajectories and that research focused on suicidal ideation is vital in mitigating suicidal outcomes and attempts (13–16). This leads to the suggestion that investigating the drivers and experiences of suicidal ideation, independent from suicidal behaviors and attempts, as well as what helped during these experiences, is a vital research direction for CIW suicide prevention (13–17).

In addition to the suggested utility of research focused on the drivers and experiences of suicidal ideation for CIW, as well as what helped during these times, recent reviews also propose the importance of a more nuanced, national direction within this research stream. These reviews indicate that driver relevance may be linked to a complex interplay between the individual and the cultural and socio-political context within which the industry functions (5–7). For example, the financial state of a nation’s construction industry or health and safety legislations will likely impact on suicide drivers such as psychosocial job adversity (e.g., level of job security, work hours) (5–7). Additionally, there are likely differences in cultural norms that impact across existing vulnerabilities among CIW populations, with some workplace cultures more likely to stigmatize mental health and suicide, resulting in decreased help seeking and offering behaviors, commonly implicated as playing a role in CIW suicide outcomes (5, 7). Ultimately, these differences across nations’ construction industries likely influence driver relevance, experiences of suicidal ideation and distress, as well as what is considered protective during these experiences. As a result, the value of researching the issue from a national perspective is apparent and allows for more contextual understandings.

The Australian Construction Industry (ACI) has been repeatedly shown to experience increased suicide vulnerability (1–3). While positive directions have been reported regarding mitigation of this vulnerability, with recent analysis suggesting a reduction in disparity between ACI suicide and that of other employed Australian men, the same research also indicates those employed in the ACI remain at elevated risk (1). As such, the continued need to create richer understandings of the nature and drivers of suicidal ideation in the ACI, to inform preventative measures, including industry specific changes to mitigate drivers, is required to ensure continued mitigation of outcomes. As a result, the purpose of this study, which to the best of the authors’ knowledge is the first of its kind, was to qualitatively explore the drivers and experiences of suicidal ideation of those employed in the ACI, what helped during this time, as well as perceptions of what the industry could do more broadly to help mitigate ACI suicidal trajectories.

## 2. Materials and methods

### 2.1. Participants

Participants were all aged over 18 years and responded to questions based on either their personal experience with suicidal ideation while employed in the ACI (*n* = 2); their experience of supporting someone experiencing suicidal ideation who was working within the ACI at the time (*n* = 8), or a combination of the two (*n* = 5). All bar one individual was employed in the ACI, or within roles closely associated with the industry (e.g., construction education and training), during the time of experience, with majority of participants remaining employed within the ACI at the time of interview. Most participants also reported long term work histories within the ACI, with many highlighting changes to their roles during their time in the industry (e.g., starting out as tradesmen/women or laborers prior to movement into current roles). The single participant not employed directly in the ACI had intimate knowledge of industry, both from personal relationships with those working within the industry, as well as direct, repeated, and regular engagement as a consultant to ACI employees, as part of their work role.

Participants were recruited using a multi-sampling method which employed both convenience and purposive strategies, to ensure those included in study were representative of the population of interest. Primary method of recruitment was distribution of study information flyers, that provided information regarding participation requirements, *via* non-for-profit construction industry suicide prevention, education, and support service MATES in Construction (MIC) South Australia, as well as promotion *via* the various MIC social media platforms. A small number of participants were recruited *via* personal and professional contacts of the authors or support workers from MIC South Australia. Careful consideration was given to the way in which these participants were approached, with said participants only informed of study existence through provision of information flyers and no further contact being made unless requested, ensuring no coercion. All potential participants registered their interest *via* email contact with lead author (ST), and all interested participants were provided further information regarding the study requirements following contact. Any questions regarding eligibility or requirements were answered prior to the scheduling of interview at MIC South Australia offices.

### 2.2. Procedures and materials

Participation was voluntary and involved partaking in a semi-structured, face-to-face interview, which was audio recorded with participant consent. A semi-structured interview guide to allow participants to provide data on key research aims, with two examples of questions posed located in Table 1 below, was developed by ST in consultation with KG, BC, and NP. No repeat interviews were required, and no participants decided not to participate once interview was scheduled. Due to the potentially distressing nature of interview content, participants were required to undertake an unrecorded Mental State Examination (MSE) with an Australian Health Practitioner Registration Agency (AHPRA) registered mental health professional (Clinical Psychologist or Mental Health Nurse) upon arrival at interview location. During this MSE participants were asked demographic questions, as well as assessed on their current mood, cognitions, perceptions, and suicidal thoughts. Based on the MSE clinical assessment, only when the registered health professional was satisfied with participant’s psychological safety to engage in interview, were participants asked to sign the consent form to participate. Following completion of interview, participants were required to engage with another unrecorded MSE, with the same AHPRA registered mental health professional, to ensure participant safety to leave interview location.

**Table 1:** Semi-structured interview question examples.

Question example
“Talking about suicide and suicidal thoughts can be tough. I appreciate you talking me through your experience of this topic. Can you tell me about any personal experience of suicidal thoughts when working in the construction industry? It can be your own (if comfortable) or your experience helping a workmate through this time?”
“Some people say that working in the construction industry has unique challenges that can be difficult for some workers to handle. What are your views on this? What, if any, are the challenges within the construction industry that you believe play a part in workers experiencing suicidal thoughts or distress?”

All interviews were conducted by ST, a male registered psychologist and early career academic, with personal experience working within the construction industry and training in qualitative research. Interviews lasted on average 38.28 min (times ranged from 25.37 to 78.20 min). Data was collected until saturation was achieved (no new themes apparent after 12 interviews as determined by ST, KG, and NP) (18). Field notes were taken during and after each interview and reflexive journaling was undertaken to monitor themes and add rigor. Interview audio recordings were transcribed verbatim, and participants were offered a chance to check and amend their transcripts to ensure information they had provided was representative of experiences and perceptions. Only 2 participants took up this offer with both making minimal changes to their transcripts.

### 2.3. Data analysis

Data were analyzed using descriptive thematic analysis, according to Braun and Clarke’s method (18). Transcripts were read and re-read by ST, with preliminary codes and themes (concepts appearing throughout the data) for each research question derived from the data. Data relating to each theme were initially organized by ST, with the aid of NVIVO software, with subsequent themes and content reviewed by KG and NP, both current academics experienced in qualitative research with clinical experience as mental health professionals, until ST, KG and NP felt key meanings in the data were expressed. An essentialist approach to analyzing the data was used, whereby the information provided by the participants was considered as direct insights into their reality. While interview questions were developed to generate information on outlined research aims and objectives, the semi-structured and open-ended nature of the interviews, as well as data analysis procedure, that sought patterns in the data prior to theorizing and that meaning was situated at the surface level, meant an inductive approach was employed. The Consolidated Criteria for Reporting Qualitative Research (COREQ) standards for reporting qualitative research were followed (19).

### 2.4. Ethical considerations

Participation was voluntary and interviews were audio-recorded with consent. To ensure participants were informed of requirements of engaging with study, they were provided a detailed participant information sheet upon expression of participation interest and any further questions were clarified by ST. As previously outlined, a Mental State Examination (MSE) was conducted pre and post interview to ensure participant safety. Participants were also provided with a list of relevant support services post interview. Additionally, all interviews were conducted by a registered psychologist, experienced in presentations of suicidality, to ensure safety within the interviews, with an extensive safety protocol developed should emotional distress arise. The study was approved by the University of South Australia Human Research Ethics Committee (protocol number 204232).

## 3. Results

Fifteen participants agreed to take part in interviews with ages ranging from 29 to 66 years of age (*M* = 45.07, SD = 10.30). All bar one participant was employed in the ACI at the time of experience, as well as at time of interview, with current roles including general managers of private construction organizations, field officers providing placement and support to ACI apprentices and occupational health, safety, and environment managers.

Despite most participants current roles now in management, as mentioned significant work histories within the ACI were reported and several participants discussed personal experiences regarding earlier roles within the ACI (e.g., when employed as tradesmen/women or laborers). Additionally, where participants discussed experiences of supporting someone experiencing suicidal ideation while employed in the ACI, the individuals refereed to were all employed in various roles within the ACI including safety and compliance management (*n* = 3), project/site management (*n* = 5), tradesman (*n* = 6), office management (*n* = 1), laborer/machine operator (*n* = 7), traffic management (*n* = 1), apprentice (*n* = 1), and small business owner (*n* = 1), resulting in broad representation of roles within the ACI. Most participants identified as being male (*n* = 10), a single participant reported migrating to Australia, and none reported being part of any minority groups (e.g., LBGTQI+ or Aboriginal and Torres Strait Islander communities). Most participants were employed by private organizations (*n* = 14), reported working more hours than receiving pay for (*n* = 13) and having engaged with an aspect of the MATES in Construction (MIC) suicide prevention and education training program (*n* = 14). Table 2 below provides an outline of participant demographics.

**Table 2:** Participant demographics.

Interview Participants			
Characteristic	*n* (%)	Range	Mean (SD)
Gender			
Male	10 (75.0)	–	–
Female	5 (25.0)	–	–
Age (years)	–	29–66	45.07 (10.3)
Education (highest level)			
Bachelor’s degree	5 (33.3)	–	–
Trade certificate/diploma	7 (46.7)	–	–
Post Graduate Diploma	2 (13.3)	–	–
Year 10 completion	1 (6.7)	–	–
Employment type			
Private Organization	14 (93.3)	–	–
Government Training Organization	1 (6.7)	–	–
Discussing own experiences	2 (13.3)	–	–
Discussing other’s experiences	8 (53.4)	–	–
Discussing own and other’s experiences	5 (33.3)	–	–
Current job role			
General manager	2 (13.3)	–	–
Safety and environment manager	5 (33.3)	–	–
Education	1 (6.7)	–	–
Project/site director or manager	4 (26.6)	–	–
Field officer (ACI apprentices)	1 (6.7)	–	–
Human resources	1 (6.7)	–	–
Insurance manager	1 (6.7)	–	–
Hours worked (weekly average)	–	35–65	51.7 (8.8)
Hours paid (weekly average)	–	35–60	39.6 (5.8)
MATES in construction training			
Yes	14 (93.3)	–	–
No	1 (6.7)	–	–

### 3.1. Topic 1. What may drive an ACI employee to experience suicidal ideation and distress?

Eight overall themes relating to what may drive the experiences of suicidal ideation for those employed in the ACI, were identified. As detailed in Table 3, these included challenges of working in the ACI, with examples being pressures and demands of job role. Additionally, themes relating to issues primarily stemming from one’s personal life, such as financial hardship, experiences of mental health challenges, trauma, or a significant adverse life event, among others, were also highlighted by participants as playing a role in driving experiences of suicidal ideation and distress.

**Table 3:** Summary of themes for drivers of ACI employee’s suicidal ideation and distress.

Themes
Challenges of working in the Australian construction industry
Relationship and family issues
Social disconnection
Personal financial hardship
Perceived lack of support (work and services)
Alcohol and drug use
Child custody/access and legal issues
Experience of mental health challenges, trauma, or a significant adverse life event

#### 3.1.1. Challenges of working within the Australian construction industry

Many participants highlighted experiences of suicidal ideation and distress in part being driven by challenges that were directly related to ACI employment. One example of these challenges was the pressures and demands relating to an individuals’ job or role. While these pressures and demands were different based on the specific job or role, those who discussed these issues, often seen as increasing in intensity in more recent times, all highlighted them as playing a significant part in driving experiences of suicidal ideation and distress.

*“The shift has gone from pressure of bullying you, you know, yelling and abuse, blah, blah, blah, carrying all of that to, um, they are nicer to you. But in the background, the pressure comes from program. So, they push you…they put you under pressure with a program. They make you sign up to a contract with a program that is that tight that if it slips, then there’s contractual penalties and potential financial penalties.”* (Participant 7, own and other’s experience).

*“You know, you have got hazardous work. We do demolition, asbestos, and civil challenges. There’s not…never ending. We do high risk. Everything we do is really considered high risk.”* (Participant 5, own and other’s experience).

Another example of a challenge stemming from ACI employment discussed by participants was work hours. Many participants highlighted how long work hours and expectations to be on site regularly, including weekends, played a significant role in driving experiences of suicidal ideation and distress while working in the ACI.

*“I’m still working like 60 h a week. But I was doing … kind of, 80, 90, 100-h weeks for a long time. Um, so, yeah, took its toll.”* (Participant 2, own experience).

Several participants also provided examples of how workplace transience and insecurity, including the shifting of workplace (e.g., work site) and the subsequent changes to co-workers, was a challenge of ACI employment that played a significant role in driving experiences of suicidal ideation and distress.

*“You know, maybe a project goes for 2 years for instance. And you might be lucky enough to stay with the same supervisor and, you know, few of the people for the 18, 2 years … 24 months, maybe longer if you are lucky. But then you know it’s going to finish. And you kind of … it’s like you are continuously changing jobs.”* (Participant 5, own and other’s experience).

Many participants also highlighted the challenge of ACI workplace cultures as driving experiences of suicidal ideation and distress, providing examples of where perceptions of industry stigma towards someone experiencing suicidal thoughts or a mental health challenge was apparent, with such beliefs suggested as limiting help-seeking behaviors or discussion of challenges.

*“I think it’s just the stigma … If you have a broken arm people can see and touch and feel it. Right? You can see it, but it gets fixed. Right? Um, diabetes, okay similar, but mental health. You cannot touch it and feel it and, um, and it’s not, it’s not accepted yet.”* (Participant 13, own and other’s experience).

*“Actually, one person in particular got me aside and wanted me to talk about it, but I just did not … I just did not feel comfortable with them, you know, discussing it. They were kind of like, you know, if you tell them something, they may be able to use it against you.”* (Participant 5, own and other’s experience).

Finally, many participants highlighted a lack of work-life balance as a challenge resulting from ACI employment that played a role in experiences of suicidal ideation and distress, including descriptions of the impact of work on ability to engage in life outside of work, such as spending time with family or being able to engage in activities important to wellbeing.

*“A lot of guys do not have work life balance. And … and if you do not have that, then that has to be, in my opinion, a contributing factor. That has to … that has to contribute towards how you are feeling. Um and for me, personally, that’s a trigger. That’s … that’s the start of a roller coaster that could go the wrong way.”* (Participant 1, own experience).

#### 3.1.2. Relationship and family issues

Many participants highlighted relationship or family issues as playing a role in driving experiences of suicidal ideation and distress, indicating challenges with a romantic partner, family, or the home environment, as apparent at the time of experience.

*“If you are home environment’s a happy, stable place. You know what I mean? You’re going to feel those vibes. You’re going to run with it. If your … home environment is, um, unhappy, depressed, quiet. Fuck it, let us not go anywhere … then, absolutely, that plays a factor.”* (Participant 1, own experience).

*“When he was at work, had an argument with his partner on the phone. And he, in his words, he was either going to jump off the side of the building or take the hoist down.”* (Participant 6, own and other’s experience).

#### 3.1.3. Social disconnection

Several participants highlighted the role social disconnection played in experiences of suicidal ideation and distress, discussing that increased loneliness, remoteness, and isolation, was apparent during these times.

*“They lived rurally, they talked about the peace and quiet of being in the country. But I just felt like that also become a bit of an isolation for them.”* (Participant 10, other’s experience).

#### 3.1.4. Personal financial hardship

Many participants highlighted that facing personal financial hardship, and the pressure that occurs because of these challenges, played a role in driving experiences of suicidal ideation and distress while working in the ACI.

*“Um, we were going through a bit of financial hardship at the time. Like … and I’ve got a good job but like we were going through some financial stuff.”* (Participant 2, own experience).

*“Yeah, financial stress. Yeah, so obviously you, ah, as somebody that wants to provide for their family, um, if then work drops off you do not know where the next job’s coming, but then you need to be able to provide that for your family, um, and then you have to have that conversation with them, going on, you know, and, you know, it’s going to be pretty rainy this week for example, and I know there’s probably not going to be as much hours because of this, and I’m not going to be paid. I think the financial bit is the huge part.”* (Participant 4, other’s experience).

#### 3.1.5. Perceived lack of support (work and services)

Several participants discussed perceptions of a lack of support as playing a role in driving experiences. This lack of support was discussed in relation to both the workplace, as well as wider social support, such as limited access to mental health services.

*“Work has not supported him in that. Um, so then that impacts. It makes him more stressed. So, something that was keeping him a little bit more sane and keeping him busy and doing things has now become stressful.”* (Participant 12, other’s experience).

*“Um and trying to get into services to get him support was probably the hardest, really, really hard thing because we could not get in anywhere. So even just getting into a GP … was hard.”* (Participant 9, other’s experience).

#### 3.1.6. Alcohol and drug Use

Several participants discussed the role alcohol or drug use played in experiences of suicidal ideation and distress, highlighting how use could lead to the presence or increase of thoughts of taking one’s life.

*“But, like, yeah, if I’d had a couple bottles of wine and I was tired, that was … that was where you are thinking about … Better off not being here.”* (Participant 2, own experience).

#### 3.1.7. Child custody/access and legal issues

Several participants highlighted challenges relating to child custody, including dealing with associated legal issues or lack of access to their children, as playing a significant role in the experiences of suicidal ideation and distress.

*“Um, he’s got two young kids. Um, and he now has not seen them since January. Um, so there’s obviously custody issues there.”* (Participant 12, other’s experience).

#### 3.1.8. Experience of mental health challenges, trauma, or a significant life event

Many participants highlighted the role of previous exposure to a mental health challenge, trauma, or a significant adverse life event, as playing a significant role in driving experiences of suicidal ideation and distress, with most participants suggesting that an occurrence of these challenges was the initial driver of experiences.

*“I…I…I think the trigger point was losing one of my…one of my mates to suicide.”* (Participant 1, own experience).

*“I know that he had some health issues that then were freaking him out, and that was just like, I think that really was the tipping point for him.”* (Participant 4, other’s experience).

### 3.2. Topic 2. What is the experience and expression of suicidal ideation and distress for ACI employees?

Four overall themes relating to the experience and expression of suicidal ideation and distress for those employed in the ACI were identified. As detailed in Table 4, suicidal thoughts was a theme identified, with participants detailing the nature of thoughts apparent during experiences. Impaired thinking was another theme identified with participants discussing limitations in some areas of cognition during experiences. Additionally, themes relating to observable expressions of suicidal distress, as well as an apparent lack of observable expression of suicidal ideation and distress in some instances, were also identified.

**Table 4:** Summary of themes for experience and expression of suicidal ideation and distress for ACI employees.

Themes
Suicidal thoughts
Impaired thinking
Observable expressions of suicidal distress
Lack of observable expression of suicidal distress

#### 3.2.1. Suicidal thoughts

Many participants described the suicidal thoughts experienced while working in the ACI, highlighting several common suicidal states that have previously been shown as central in leading one to think of taking their life. For example, several participants discussed thoughts of hopelessness, highlighting their presence in experiences of suicidal ideation and distress while working in the ACI.

*“Like, you know, now it’s hopeless. And like all the same stuff that you just hear about. You know, it’s hopeless. Um, I do not know what to do, you know. I cannot remember exactly what he said, but it was … it was words to that effect.”* (Participant 11, other’s experience).

Many participants also discussed thoughts of being trapped or overwhelmed and how these perceptions were a significant component of thoughts of suicide while working in the ACI.

*“That situation … to get me away from that situation, I was either going to take my life or I was going to quit.”* (Participant 11, other’s experience).

*“And you feel like you are suffocating. Like, you know, how the fuck am I going to get out of all this?”* (Participant 7, own and other’s experience).

Additionally, thoughts of burdensomeness, including how one thought family or friends would be better off without them alive, were also discussed, with many participants highlighting how these perceptions influenced one’s thinking regarding ending one’s life during experiences.

*“And then, um, yeah, the most logical thing for me, like I was just in this dead set logical mindset that it was better for myself and my family if I wasn’t around.”* (Participant 2, own experience).

Several participants also discussed the fluctuating nature of suicidal thoughts, including how there were changes in prevalence and severity of thoughts, often dependent on the presence of drivers.

*“So, um … so yeah, to answer … answer your question, it was not a daily occurrence. It fluctuated depending on the load that I was under.”* (Participant 7, own and other’s experience).

*“You know, you would have random thoughts about it. But then it got stronger. Like you’d think about it every day.”* (Participant 1, own experience).

Several participants discussed suicidal plans, including intentions of how to end their life and that if means had been available the likelihood of outcome occurring would have increased, that were apparent during experiences of suicidal thoughts and distress while working in the ACI.

*“If … if there was the means and method and it solved the problem and you did not have to feel anything anymore, you could probably do it pretty quickly if … if you were in that, you know … if you had that all-in front of … of you.”* (Participant 5, own and other’s experience).

#### 3.2.2. Impaired thinking

Several participants discussed the presence of impaired thinking during experience of suicidal ideation and distress while working in the ACI, highlighting inability to think outside of suicidal distress, as well as decreased logic or judgment.

*“Um, you do start getting those thoughts on … because you are … because you are so caught up in yourself, you do not … you do not understand the repercussions it’s going to have.”* (Participant 1, own experience).

#### 3.2.3. Observable expressions of suicidal distress

Many participants discussed how the experience of suicidal distress was expressed and provided examples from experiences. For example, several participants highlighted how during experiences total disengagement with, or decreases in motivation to perform, self-care activities were apparent.

*“Love me fishing … I know that’s my outlet. I got to a point where I could not even be fucked going fishing.”* (Participant 1, own experience).

Additionally, several participants highlighted that significant reductions or challenges with sleep were apparent during experiences of suicidal ideation and distress, suggesting this was an observable expression of this challenging time.

*“Waking up in the middle of the night and waking up repeatedly. So, I start off a little bit and then it repeated and then they were longer periods of being awake during the middle of the night. Some nights I had not slept for 2 days kind of thing. You know, and you feel terrible.”* (Participant 5, own and other’s experience).

Changes in workplace behavior during experiences of suicidal ideation and distress, including increased absenteeism and changes in appearance or regular presentation, was also highlighted by participants as an observable expression of suicidal distress.

*“I saw these people and I was going – saw they were normally like for a while, and then they just changed. There was this kind of weird. It was odd. It was like a feeling that they are not – that’s not their normal behavior, their normal look.”* (Participant 5, own and other’s experience).

*“It contributed to significant absenteeism from work and something that I needed to manage, um, with a level of empathy that I would probably not give to most people.”* (Participant 10, other’s experiences).

Several participants discussed the presence of self-harm and suicidal attempts as observable expressions, discussing presence, and at times increases, of these potentially life-threatening behaviors during experiences of suicidal ideation and distress while working in the ACI.

*“Um, initially he was scratching … his arm and then it just got progressively more and then it got worse and then he was cutting himself.”* (Participant 9, other’s experience).

Several participants discussed increases in interpersonal challenges, regarding workplace colleagues, as well as those from personal life, as observable expressions, highlighting the presence of these conflicts, suggested as different from normal behaviors, during experiences of suicidal ideation and distress while working in the ACI.

*“I like remember going up on the deck and say, hey man, like the task you are doing here, like, you are supposed to have some flags up, you know. You’re supposed to have like an exclusion zone, so – so people cannot come into your work area cause he’s doing dangerous stuff, you know. And he is like, fuck this. He just grabs like this 44 gallon and threw it down stairs. You know, and then we went for a walk. And that’s when he sort of started explaining everything to me.”* (Participant 11, other’s experiences).

#### 3.2.4. Lack of observable expression of suicidal ideation and distress

In contrast many participants discussed the lack observable expression of suicidal distress, highlighting that in many experiences minimal communication of challenges experienced, *via* either verbal expression, changes to behavior or help seeking behaviors, was apparent.

*“Some people you can tell. You think, oh jeez, he fires up easy or … or what the hell, he’s had a … he’s had a big weekend and he’s … he’s picking fights with people on site on a Monday. And you can sort of pick maybe a little bit, but, you know, this guy I just had no idea.”* (Participant 7, own and other’s experience).

### 3.3. Topic 3. What helped during these experiences of suicidal ideation and distress and what can the ACI do to mitigate these outcomes?

Six themes relating to what helped during experiences of suicidal ideation and distress, as well as what can be done by the ACI to help mitigate these outcomes, were identified. As detailed in Table 5, participants highlighted factors related to the industry that were seen as helpful or protective during experiences, including the presence of colleague and managerial support, as well as the industry specific suicide prevention, education, and support service MATES in Construction. Additionally, participants highlighted factors from their personal lives that were seen as helpful or protective during experiences, with engagement with non-work activities and social support, as well as personal skills and knowledge relating to suicide and mental health, both discussed. Additionally, participants highlighted the need for high level industry integration and engagement with prevention, education, and support programs, as well as changes to work hours and expectations, as important areas that require industry focus to mitigate experiences of suicidal ideation and distress for those employed in the ACI.

**Table 5:** Summary of themes for what helped during experiences and what the ACI can do to mitigate outcomes.

Themes
Colleague and managerial support
MATES in Construction
Engagement with non-work activities and social support
Personal skills and knowledge relating to suicide and mental health
High level industry integration and engagement with support programs
Work hour and expectations changes

#### 3.3.1. Colleague and managerial support

Many participants discussed the important role played by workplace colleagues and managers in helping and supporting during experiences of suicidal ideation and distress while working in the ACI.

*“They needed the support of their work colleagues as a friend, but also as their manager to get them through, because possibly if they did not have the work side of things, we do not know where they could have ended up.”* (Participant 10, other’s experiences).

#### 3.3.2. Mates in construction

Many participants highlighted the significant role industry specific suicide prevention education and support service MATES in Construction played in assisting during experiences of suicidal ideation and distress while working in the ACI. This assistance was discussed in two ways, being reduction of stigma, therefore leading to reduced perceptions of judgment when seeking help for suicidal ideation and distress, as well as *via* the practical support services the organization provides, such as a 24 h helpline.

*“Um, so MATES in Construction, the function they play is not just about destigmatizing that discussion, but it’s providing comfort that there is a number that is immediately going to be able to help you.”* (Participant 2, own experiences).

#### 3.3.3. Engagement with non-work activities and support services

Several participants discussed the important role played by engaging in activities outside of work and social support, whether that be friends and family, or mental health services, in helping during experiences of suicidal ideation and distress while working in the ACI.

*“You have to have an outlet outside of home, work. And it has to be about you.”* (Participant 1, own experience).

*“And it got to a point where I ended up seeking professional help. My GP, um, referred me to a psychologist, um, who I saw off and on for about 6 months. Um, and they definitely helped.”* (Participant 7, own and other’s experience).

#### 3.3.4. Personal skills and knowledge relating to suicide and mental health

Several participants discussed how they felt that personal learnings and knowledge regarding suicide and mental health helped during experiences, with such skills and knowledge generated by lived experience or engagement with support services.

*“Second time around, I think, because I had the tools and I had the understanding, I was further educated in mental health. I think I — I stopped that progression.”* (Participant 1, own experience).

#### 3.3.5. High level industry integration and engagement with support programs

Many participants discussed the need for increased high level industry integration and engagement with support programs to mitigate experiences of suicidal ideation and distress, with many highlighting the importance of those in high level positions and organizations to incorporate support programs that provide education and training focused on drivers of suicidal ideation and distress into their workplaces.

*“Yeah, and what can we do to help, whether it’s a health programs, and you do target, you know, depression on, you know, you do depression, you could do drugs and alcohol, you could do gambling, you know, you could do your finances, offer budgeting, um, yeah, there’s so many snippets that could form a huge big program, that then everyone on, on a big project can do.”* (Participant 4, other’s experiences).

#### 3.3.6. Work hour and expectations changes

Many participants discussed the need for changes to work hours and expectations surrounding these work hours, which are common within the industry, to mitigate experiences of suicidal ideation and distress for those employed in the ACI.

*“So, 5 day working weeks or limit hours at least. And you know, give people some time for life.”* (Participant 11, other’s experiences).

*“So I, personally, believe the industry, within themselves, should … and … and obviously it’s going to be organization by organization structure. But as a whole, I think they need a good hard look. Um, and also the … the support behind that is, like, say long weekends. Fucking shut the shop. Shut the site.”* (Participant 1, own experiences).

## 4. Discussion

This study explored the drivers and experiences of suicidal ideation and distress for those employed in the ACI, what helped during this challenging time, and what the ACI can do to mitigate experience of these challenges and further suicidal trajectories. Information was generated from perspectives of workers who have experienced it themselves, or supported someone in the industry through this challenging time.

Several drivers of suicidal ideation and distress highlighted by participant accounts were the result of challenges arising from ACI employment. Participants highlighted challenges with job pressures and demands, sometimes seen as increasing in more recent times, in part drove experiences. Participants also highlighted that the work hours undertaken and regularly required in the ACI played a role in driving experiences. Additionally, participant accounts highlighted challenges with workplace transience and insecurity, including the shifting of workplace (e.g., work site) and the subsequent changes to co-workers, as another significant driver due to ACI employment. These findings support previous investigations of CIW and ACI populations which suggests that industry drivers, such as psychosocial job adversity, are central in experiences of suicidal ideation and distress (6–8). However, while supporting previous research, the current study provides richer information on the specific workplace challenges that may be relevant to experiences. While it is acknowledged that addressing any psychosocial job adversities would likely prove beneficial in mitigating experiences, the current study’s findings do highlight focused efforts on relieving job pressure and demands, work hours and workplace transience and insecurity, highlighted in this study as the most central industry drivers of suicidal ideation and distress experiences, may prove most productive. Furthermore, with repeated demonstration of the impact workplace challenges have on suicidal ideation and distress for this population, the need for the ACI to mitigate impacts where possible, is reinforced (6–8).

Another industry challenge highlighted by this study was workplace cultures that stigmatize suicide and mental health, potentially limiting help seeking and help offering behaviors. This is a significant concern for the ACI as many suicide prevention approaches in the area are reliant on people engaging in help seeking behaviors, an unlikely occurrence if one perceives they will be judged or dismissed by colleagues when doing so (20, 21). Previous research in the area has suggested this stigma may be the result of the ACI’s increased adherence to traditional masculine norms (3). Despite this suggestion, more recent research indicates only certain domains of traditional masculine norms, such as self-reliance, may play a role (3, 6, 8). Further research is needed regarding if, how and why the ACI stigmatizes suicide and mental health, to understand how to best create industry culture change, or at worst mitigate its impact on help seeking behavior. Personal attitudes and stigma towards suicide and mental health, beyond industry cultures, may also play a role in limiting help seeking or increasing adherence to self-reliance behaviors. While these personal attitudes and stigma will require focus from broader social interventions and messaging, the current study highlights the importance of industry engagement with tailored preventative programs that are shown to address and de-stigmatize both industry and personal beliefs regarding suicidal behavior and mental health, with such programs affirming that it is ok to seek help (21). Additionally, engagement with programs or services that provide training to the industry so the workforce can proactively offer support to colleagues, rather than waiting for distressed people to reach out to them, is another important step the ACI should consider based on these findings (21).

Participants also highlighted the lack of work-life balance resulting from ACI employment has on driving experiences of suicidal ideation and distress while working in the ACI. This is a similar finding to that of a global review of drivers of suicide for CIW that stressed the potential crossover between industry and external drivers (7). This finding suggests the need for the ACI to understand the negative impact that challenges arising from industry employment can have on one’s life outside of work and subsequent wellbeing. Furthermore, the finding emphasizes the need for ACI changes to be made were possible, such as to work hours and requirements, to mitigate development of, and impacts on, suicidal ideation and distress.

Participant accounts also highlighted several other potential drivers of suicidal ideation and distress that can be viewed as external to the ACI. Relationship and family issues, whether with a romantic partner, friend, or family member, have long been known to drive suicidal trajectories, particularly in later stages, and the current study’s findings highlight the significant role they can play in earlier trajectories of suicide for those employed in the ACI (3, 7). Similarly, personal financial hardship, including the pressure that is experienced because of these challenges, as well as child custody and legal issues, drug and alcohol use and experience of a mental health challenge, trauma, or significant adverse life event, have also been shown as present prior to suicide in ACI employees. However, until the current study, these drivers have not been shown as relevant in early stages of the suicidal trajectory such as suicidal ideation (3). While many of these drivers are seen as best addressed through wider social changes and support, the clear role the ACI and preventative groups can play in mitigating influence of these drivers is also apparent (5, 7). By ACI and preventative groups understanding said driver relevance and educating the industry on the role they may play in early in suicidal trajectories, including that early identification of such challenges and how they may influence someone’s suicidal trajectory is important, increased driver mitigation is viable.

Participants also highlighted a perceived lack of support, as well as social disconnection, as drivers of experiences. This finding is important for preventative groups to understand how they can potentially influence presence of suicidal ideation and distress by continued education around the importance of social connection, seeking support, and availability of services during these challenging times. Additionally, this finding highlights the role the ACI can play in mitigating the influence of these drivers. Increased social disconnection is potentially circumvented through workplace support and engagement with those experiencing such challenges, and as mentioned previously training and education so the workforce has the skills to support those in need is a recommended action. Similarly, wider ACI engagement with, and encouragement of available support service use, may also lead to mitigation of these drivers’ by reducing perceptions that there is a lack of social support available (21).

Participant accounts highlighted that there are similarities in experiences of suicidal ideation and distress for those employed in the ACI, with many others outside of the industry. Many participants highlighted thoughts of hopelessness, being trapped/overwhelmed or burdensomeness were present during experiences. These psychological constructs have been identified as central in suicidal trajectories in many of the highly regarded theories and models of suicidality such as the Interpersonal Theory of Suicide (ITP) and the Integrated Motivational Volitional Model of Suicidal Behavior (IMV) (22, 23). The presence of these thoughts is an interesting finding and adds further support to their centrality within suicidality processes, including for those employed in the ACI (22, 23). Additionally, these findings add support to their potential role in earlier stages of suicidal trajectories, such as development of suicidal ideation (22, 23). It is important that ACI prevention, education, and support groups are aware of the centrality of such thoughts in one’s experiences and that processes to mitigate or restructure such perceptions are likely vital in interrupting an ACI employee’s suicidal trajectory. Several participants also highlighted that during these experiences, fluctuations of thoughts and presence of suicidal planning were apparent. With fluctuations in thought prevalence and severity reported as dependent on driver presence, it suggests not only to the importance of ACI mitigation of identified drivers, but also that when drivers are present and cannot be influenced, engagement with industry intervention, education and support services is vital (6, 7). Similarly, the presence of suicidal planning and impaired thinking reported by several participants again highlights the importance of ACI engagement with such services. Previous research has shown that for many experiencing suicidal ideation the speed in which one can transition from ideation to attempt or outcome, can be quick (24). Therefore, opportunities to intervene can be limited, particularly if an inability to think objectively is apparent (24). Repeated ACI engagement with prevention programs that create knowledge of the availability of support during these critical times, encourage help-seeking, and upskill the workforce in suicide prevention skills, is vital in mitigating suicidal trajectories, particularly for those who are experiencing impaired thinking or engaging in suicidal planning (21).

Participant accounts highlighted that in many of the experiences of suicidal ideation and distress discussed, there were observable expressions that someone was experiencing these challenges. Changes in desire to undertake or engage with usual self-care activities, impacts to sleep, variations in usual workplace behavior, engaging in self-harm behaviors or attempting suicide, as well as experiencing an increase in interpersonal challenges (home and workplace) were all highlighted as present in several accounts. This is vital information as it informs both the industry and prevention, support, and education programs that these are potential indicators that someone is experiencing suicidal ideation and distress, and that support is required. Additionally, education regarding presence of these challenges not only informs when one may need to offer help to a colleague but also creates knowledge that these may be early indicators that oneself may be struggling and engagement with support services is vital (25). However, despite many participant accounts highlighting the presence of observable expressions of suicidal ideation and distress during experiences, many also highlighted their inability to identify such expressions. This finding is in line with previous research that suggests adherence to the traditional masculine norm domain of self-reliance may limit ACI overt expression of suicidal ideation and distress (7, 8). Similarly, it aligns with suggestions of potential impacts both personal and ACI stigma towards suicide and mental health have on expressions of these challenges (7, 8). With this lack of observable expression potentially impacting ability for workplace identification, findings again highlight the importance of industry engagement with tailored preventative programs that destigmatize suicide and mental health, encourage help-seeking behaviors and educate regarding limitations of self-reliance behaviors (21).

Participant accounts highlighted several areas, from both within the industry and from personal life, that helped during these experiences. These are important findings to inform the industry, as well as support, education, and prevention programs, on what may be required by those within the ACI experiencing suicidal ideation and distress. Colleague and managerial support were reported as helpful, and this aligns with recent research in broader Australian male populations of the protective role social support plays in male experiences of suicidal ideation (26). It has been suggested that social support may increase men’s perceptions of self-worth and value, an important protector against common perceptions regularly seen in male suicidal experiences, including in the current study, such as perceptions of worthlessness (27). As such, this highlights the importance of not only those experiencing suicidal ideation and distress leveraging support from those around them, but also the vital role those within the workplace can play. Personal skills and knowledge relating to suicide and mental health, as well as the industry prevention, support, and education program MATES in Construction (MIC), including the availability of their 24 h support line and stigma reduction activities, were also emphasized as helpful during experiences. While personal skills and knowledge may be developed outside of MIC, findings highlight the vital role MIC plays, from education to active support, and that continued industry engagement with such programs is vital. They also reinforce the importance of social support availability, whether from colleagues or broader community services, in protecting against such experiences within the sector (28). Participant accounts also highlighted the relevance of personal factors or behaviors from their personal lives that were helpful during experiences including engagement with non-work activities and support services, external to MIC. The importance of engagement with these activities and services is understandable and emphasizes the need for industry consideration of structural changes, such as to work hours and requirements, to allow such engagement.

Participants also discussed what the ACI could do to help mitigate experiences and suggested the need for high level industry engagement with, and integration of, suicide support, education, and prevention programs. While programs such as MIC are apparent within the ACI, there are organizations who do not engage with MIC, or other services of this kind (29). With research showing the positive impact these programs can have on suicidal trajectories, employee wellbeing and industry finances, as well as being called for by participants in the current research, the need for wider engagement is recommended for the ACI (1, 21, 30). Finally, participants discussed the importance of changes to work hours and expectations from within the industry. As highlighted by previous research, as well as the current study, work hours are seen as placing a significant burden on those within the industry and likely play a role in driving suicidal ideation and distress experiences (3, 7, 8, 31). With repeated evidence suggesting the impact these work expectations and requirements can have on those employed within the ACI, the need for the industry to consider changes is vital.

### 4.1. Strengths and limitations

This is the first study of its kind to utilize personal accounts of experiences from those with intimate and extensive knowledge of the ACI, to create better understandings of drivers and experiences of suicidal ideation and distress while employed in the industry, as well as what helped during these challenging times. The importance of allowing lived experience voices to inform understandings is vital in the development of effective prevention strategies that are required to mitigate future experiences (32). The multi-method sampling strategy, employing both convenience and purposive strategies, led to a variety of experiences being included, as well as accounts of experiences from a variety of job roles within the ACI, such as managers as well as laborers, providing a broad understanding of theme relevance across the industry. Additionally, because of the sampling strategy participant inclusion was of only those with intimate knowledge of the ACI, reducing presence of irrelevant or anecdotal information. However, the sample can be seen as having limitations, including that all participants were aged 29 years or older indicating a lack of representation of some aspects of the ACI, such as apprentices. While one participant was currently employed as a field officer supporting apprentices, this individual did not discuss experiences pertaining to this cohort. Research has indicated that this cohort may be at further increased vulnerability to suicide trajectories and one of the key reasons for this vulnerability, workplace bullying, was not a theme identified in the current study (33). Additionally, not everyone in the sample discussed personal experiences, potentially limiting intimate understandings of what drove thoughts or behaviors in individual being discussed. Finally, the sample may have been somewhat biased towards the utility of MIC, with all but one participant having previously engaged in MIC training.

### 4.2. Future directions

Future research of a similar methodology using a broader range of CIW is recommended (e.g., inclusion of apprentices). As mentioned, previous research has highlighted workplace bullying as a significant driver of suicidal ideation and trajectories for construction industry apprentices (33). This suggests there may be differences regarding drivers and experiences of suicidal ideation and distress, as well as what helped during these times, dependent on role and position within the industry. By generating understanding of what is relevant, dependent on role and position would allow for more nuanced and targeted approaches to driver mitigation and support. Additionally, using similar methodologies across various nations construction industries will develop richer understandings of drivers and experiences that are relevant across construction industry settings, as well as what cultural and socio-political differences, within which the industry functions, can influence said drivers and experiences. Again, understandings generated by this research can assist in more effective driver mitigation and support, providing useful information on areas all construction industries need to place focus, as well as what issues may arise dependent on an industries current cultural and socio-political climate.

Future research focused on deeper understanding on whether those in the ACI are more vulnerable to known drivers of suicidal trajectories, such as social disconnection or family issues, because of industry-specific factors, would prove beneficial. Minimal research has investigated the impact of industry drivers on those regularly perceived to be independent of the industry (e.g., child custody issues) despite previous research suggesting a likely interplay between industry and personal drivers. Additionally, further research is needed regarding how and why the ACI stigmatizes suicide and mental health. While previous research has alluded to the role adherence to self-reliance norms plays in impeding those within the ACI from seeking help, future research that focuses on whether there are other social constructs that limit help-seeking behaviors, whether adherence to these constructs is primarily the result of ACI culture or personal beliefs, and how best to mitigate their influence, is required (8).

## 5. Conclusion

Despite regular reporting of CIW increased vulnerability to suicide, little research has been undertaken to understand what may drive these outcomes. Furthermore, little generation of knowledge regarding drivers and experiences of early suicidal states, suggested by scholars as an important area of investigation to mitigate further suicidal trajectories, has occurred (1, 3, 8, 14, 15). While this gap in the literature requires rectification on a global level, research has also highlighted the importance of national approaches to investigation, with suggestion cultural and socio-political factors, within which the industry functions, play a role in suicidal trajectories (5, 7). As a result, this study, the first of its kind to the best of authors knowledge, investigated suicidal ideation and distress in the Australian Construction Industry (ACI) by thematically analyzing lived experience accounts of what drove and what it was like to experience suicidal ideation and distress while working in the ACI, as well as what helped during these challenging times.

The findings highlight several ACI drivers of suicidal ideation and distress, as well as those from personal lives, which can potentially be mitigated by industry changes and focused prevention strategies. Results suggest similarities with previous understandings regarding descriptions of suicidal thoughts for those within the ACI, as well as highlighting several expressions of suicidal ideation and distress that were apparent during experiences. However, findings also indicate the challenges faced by the ACI in identifying those struggling with these challenges, due to a lack of recognizable outward expression, reinforcing the importance of driver mitigation, as well as de-stigmatization of suicide and mental health to encourage help seeking behaviors. Finally, findings indicate several factors, from both the industry and personal lives, that helped during experiences of suicidal ideation and distress while working in the ACI, as well as what the ACI can do to mitigate future experiences, with several recommendations made to create a better environment, as well as effective support systems, for those experiencing these challenges.

## Data availability statement

The datasets presented in this article are not readily available because of ethical restrictions. Requests to access the datasets should be directed to simon.tyler@mymail.unisa.edu.au.

## Ethics statement

The studies involving human participants were reviewed and approved by Ethics Committee of the University of South Australia. The patients/participants provided their written informed consent to participate in this study.

## Author contributions

Conceptualization was undertaken by ST, KG, BC, and NP. Methodology was developed by ST, KG, and NP with formal analysis developed and undertaken by ST, KG, and NP. Writing completed by ST, with review and editing by KG, BC, and NP. Supervision provided by KG, BC, and NP. All authors contributed to the article and approved the submitted version.

## Funding

The research is funded by MATES in Construction National and South Australia, Return to Work South Australia and the inaugural Allison Milner Memorial PhD Scholarship.

## Conflict of interest

The authors declare that the research was conducted in the absence of any commercial or financial relationships that could be construed as a potential conflict of interest.

The reviewer VR declared a shared reference group with the authors ST, NP to the handling editor.

## Publisher’s note

All claims expressed in this article are solely those of the authors and do not necessarily represent those of their affiliated organizations, or those of the publisher, the editors and the reviewers. Any product that may be evaluated in this article, or claim that may be made by its manufacturer, is not guaranteed or endorsed by the publisher.
